# Similar patterns of clonally expanded somatic mtDNA mutations in the colon of heterozygous mtDNA mutator mice and ageing humans

**DOI:** 10.1016/j.mad.2014.06.003

**Published:** 2014-07

**Authors:** Holly L. Baines, James B. Stewart, Craig Stamp, Anze Zupanic, Thomas B.L. Kirkwood, Nils-Göran Larsson, Douglass M. Turnbull, Laura C. Greaves

**Affiliations:** a Centre for Brain Ageing and Vitality, Institute for Ageing and Health, The Medical School, Newcastle upon Tyne NE2 4HH, UK; b Max Planck Institute for Biology of Ageing, Cologne, Germany; c Centre for Integrated Systems Biology of Ageing and Nutrition, Institute for Ageing and Health, Newcastle University, Campus for Ageing and Vitality, Newcastle Upon Tyne NE4 5PL, UK; d Wellcome Trust Centre for Mitochondrial Research, Institute for Ageing and Health, Newcastle University, Newcastle upon Tyne NE2 4HH, UK

**Keywords:** Mitochondria, Ageing, Colon, MtDNA, Mouse

## Abstract

Clonally expanded mitochondrial DNA (mtDNA) mutations resulting in focal respiratory chain deficiency in individual cells are proposed to contribute to the ageing of human tissues that depend on adult stem cells for self-renewal; however, the consequences of these mutations remain unclear. A good animal model is required to investigate this further; but it is unknown whether mechanisms for clonal expansion of mtDNA mutations, and the mutational spectra, are similar between species. Here we show that mice, heterozygous for a mutation disrupting the proof-reading activity of mtDNA polymerase (*PolgA*^*+/mut*^) resulting in an increased mtDNA mutation rate, accumulate clonally expanded mtDNA point mutations in their colonic crypts with age. This results in focal respiratory chain deficiency, and by 81 weeks of age these animals exhibit a similar level and pattern of respiratory chain deficiency to 70-year-old human subjects. Furthermore, like in humans, the mtDNA mutation spectrum appears random and there is an absence of selective constraints. Computer simulations show that a random genetic drift model of mtDNA clonal expansion can accurately model the data from the colonic crypts of wild-type, *PolgA*^*+/mut*^ animals, and humans, providing evidence for a similar mechanism for clonal expansion of mtDNA point mutations between these mice and humans.

## Highlights

• Colonic crypts with mitochondrial dysfunction accumulate with age in *PolgA*^*+/mut*^ mice.
• Mitochondrial dysfunction is caused by clonally expanded mtDNA point mutations.
• The mutations are random and their expansion is not subject to selective constraints.
• Colonic crypts of aged humans have a similar mtDNA mutation spectrum and phenotype.
• *PolgA*^*+/mut*^ mice are a good model to study mitochondrial dysfunction in ageing colon.

## 1 Introduction

Ageing is a stochastic process characterised by a decline in the replicative and regenerative processes within tissues, resulting in impaired tissue homeostasis and increased susceptibility to disease and eventually death (Kirkwood, 2005). Damage to the mitochondrial DNA (mtDNA) resulting in respiratory chain dysfunction has been proposed to be a significant contributor to the ageing phenotype (Bratic and Larsson, 2013, Larsson, 2010, Linnane et al., 1989).

The human mitochondrial genome is a covalently closed molecule of ∼16.5 kb, encoding 13 proteins, 2 rRNAs (12s and 16s) and 22 tRNAs (Anderson et al., 1981). The 13 mtDNA encoded proteins form essential subunits of the oxidative phosphorylation system (OXPHOS), and in the absence of mtDNA expression the OXPHOS system breaks down (Larsson et al., 1998). MtDNA is present in multiple copies in the mitochondrial matrix where it is compacted to form nucleoprotein complexes, with the aid of mitochondrial transcription factor A (TFAM) (Kukat and Larsson, 2013).

MtDNA has a ∼10 fold higher mutation rate than the nuclear DNA thought to be primarily due to endogenous replication errors mediated by mtDNA polymerase (Zheng et al., 2006) and possibly also unrepaired oxidative lesions. Special mechanisms exist to minimise maternal transmission of mutated mtDNA (Fan et al., 2008, Freyer et al., 2012, Krakauer and Mira, 1999, Stewart et al., 2008b); however humans still frequently inherit low level mtDNA heteroplasmy (He et al., 2010, Payne et al., 2013). Given the multi-copy nature of mtDNA within cells, and the fact that most mtDNA point mutations are recessive, clonal expansion of mtDNA mutations must occur until a critical threshold level is reached that impairs the respiratory chain. Data from computational models (Coller et al., 2001, Elson et al., 2001, Taylor et al., 2003) and mitochondrial mutation assays (Coller et al., 2005) suggest that mtDNA mutations arise early in life and subsequently expand to levels high enough to cause respiratory chain dysfunction. This defect can be readily identified by the absence of histochemical staining for cytochrome *c* oxidase (COX) activity (Old and Johnson, 1989), and this serves as an excellent biomarker of mtDNA defects in somatic tissues. Although the mechanism of clonal expansion is not definitely known, there is some evidence that random genetic drift may be sufficient (Elson et al., 2001, Kowald and Kirkwood, 2013, Taylor et al., 2003).

In a number of human replicative tissues that depend on adult stem cells for self-renewal (e.g. colon, stomach, small intestine, liver and pancreas), somatic mtDNA mutations have been found to clonally expand and cause an accumulation of COX deficient cells with age (Fellous et al., 2009, McDonald et al., 2008, Taylor et al., 2003). We have previously shown that in the ageing human colon somatic mtDNA point mutations occur at random and their expansion is not subject to selective constraints (Greaves et al., 2012). The functional consequences of mtDNA defects in human stem cells remain largely unknown, partly due to a lack of robust stem cell markers but also due to the static nature of human tissue samples available at only one time point. An animal model which shows similar evidence of age-related clonally expanded somatic mtDNA mutations is therefore likely to prove valuable in enabling more detailed phenotyping of the functional consequences of mtDNA defects in stem cell populations, particularly as more robust stem cell markers are available for mouse tissues (Barker et al., 2007, Itzkovitz et al., 2012).

We have previously shown that aged wild-type mice display significantly lower levels of COX deficient colonic crypts (∼1.5% at 36 months old) compared to aged humans (∼15% over the age of 70) (Greaves et al., 2011). If random genetic drift is the mechanism by which COX deficiency occurs in mouse colonic crypts, it may be that the shorter lifespan of the mouse does not allow enough time for the threshold mutation load to be reached (Kowald and Kirkwood, 2013). Therefore normal ageing mice are not suitable for the investigation of mtDNA defects in ageing stem cells. The mtDNA mutator mouse (*PolgA*^*mut/mut*^) has a homozygous mutation (D257A) in the proof-reading domain of the catalytic subunit of the mtDNA polymerase, which results in increased levels of mtDNA mutations and a severe premature ageing phenotype. This mouse model has established a causal relationship between the accumulation of mtDNA point mutations and ageing (Kujoth et al., 2005, Trifunovic et al., 2004). The *PolgA*^*mut/mut*^ mice accumulate mtDNA mutations to a significantly higher frequency than seen in normal human ageing (Khrapko et al., 2006); however, mice heterozygous for the D257A mutation (*PolgA*^*+/mut*^) accumulate only a moderate level of mtDNA mutations (Kraytsberg et al., 2009). Furthermore, they demonstrate age-related respiratory chain deficiency in the heart and duodenum (Vermulst et al., 2008), similar to humans.

Here we compare the frequency of COX deficient colonic crypts in the *PolgA*^*+/mut*^ mice with that of colonic epithelium samples from ageing humans. We show that the *PolgA*^*+/mut*^ mice accumulate COX deficient crypts in an age-dependent manner, and that the mutations causing the COX deficiency are clonally expanded mtDNA point mutations. These mutations are similar in location and pathogenicity to those detected in the ageing human colon and also appear to lack selective constraints. Computer simulations suggest that increasing the mtDNA mutation rate within a random genetic drift model of clonal expansion can result in the observed phenotypes in the *PolgA*^*+/mut*^ mouse, suggesting a similar mechanism for clonal expansion of mtDNA point mutations in the colonic crypts of this mouse model and humans.

## 2 Materials and methods

### 2.1 Mouse strains and colon samples

Mitochondrial mutator mice (*PolgA*^*mut/mut*^) were generated that had a knock-in missense mutation (D257A) in the second endonuclease proofreading domain of the *PolgA* catalytic subunit of the mtDNA polymerase (Trifunovic et al., 2004). Colon samples were collected from an ageing series of 10 heterozygous mutator mice (*PolgA*^*+/mut*^) (aged 18–81 weeks old).

### 2.2 COX/SDH histochemistry and DNA isolation from single colonic crypts

Colon samples were collected, mounted for sectioning and frozen in isopentane previously cooled to −160 °C in liquid nitrogen. Cryostat tissue sections (10 μm) were cut on to glass slides and dried at room temperature for 1 h. A standard histological stain (Haematoxylin and Eosin) was performed on 10 μm colon sections to examine normal tissue morphology. Dual colour histochemistry was performed to determine the magnitude of COX deficient crypts and for subsequent laser micro-dissection and DNA extraction. Sections were incubated in COX medium (100 μM cytochrome C, 4 mM diaminobenzidinetetrahydrochloride and 20 μg ml^−1^ catalase in 0.2 M phosphate buffer pH 7.0) at 37 °C for 25 min. Sections were washed in phosphate buffered saline (PBS) (3 × 5 min) and then incubated in SDH medium (130 mM sodium succinate, 200 μM phenazinemethosulphate, 1 mM sodium azide, 1.5 mM nitrobluetetrazolium in 0.2 M phosphate buffer pH 7.0) at 37 °C for 35 min. Colon sections were washed in PBS (3 × 5 min) and dehydrated through graded ethanol (70%, 95% and 2 × 100%) and two concentrations of Histoclear™ (National Diagnostics, Atlanta, USA) and mounted in DPX. The percentage of COX deficient colonic crypts was identified in transverse colon sections at multiple different levels and approximately 500 crypts were examined per tissue sample.

For laser micro-dissection frozen sections of colon tissue (15 μm) were mounted on polyethylenenaphthalate slides (Leica Microsystems). Sections were exposed to dual colour histochemistry, as described above, and sections were air-dried after dehydration through graded ethanol. Single COX positive and COX negative colonic crypts were cut using Zeiss PALM micro-dissection system into sterile 0.5 ml PCR tubes, samples were centrifuged at 14,000 rpm for 15 min and lysed in standard lysis buffer (Taylor et al., 2003).

### 2.3 MtDNA sequencing of individual colonic crypts

The single crypt lysate (see above) was used as the DNA template to establish the complete sequence of the mouse mitochondrial genome from the single micro-dissected crypts. A single stage PCR reaction was employed that involved amplification of the mitochondrial genome using 30 pairs of forward and reverse primers to generate overlapping fragments of 1–1.2 kb spanning the whole mouse mtDNA genome. The primer pairs were tagged with M13 sequence to enable sequencing of the PCR products with a universal M13 primer that is designed to anneal at 58 °C. The DNA lysate was taken to a 1:10 dilution and PCR reactions were implemented in 37.5 μl volumes using a mastermix comprising 1× LA PCR buffer (Mg^2+^) (Takara Bio Inc.), 0.2 mM dNTPs, 0.9 μM primers, 5 U LA Taq DNA Polymerase (Takara Bio Inc.) and 3.75 μl of single cell lysate (1:10 dilution). Reaction conditions were 94 °C for 1 min followed by 35 cycles of 94 °C for 20 s, 58 °C for 20 s and 72 °C for 2 min. The optimal final extension was at 72 °C for 2 min.

PCR products were taken to a 1:2 dilution and purified using TSAP (Promega) to remove excess primers, and samples were sequenced using BigDye v3.1 terminator cycle sequencing chemistries on an ABI3130xl Genetic Analyser (Applied Biosystems). The sequence for each crypt was aligned to the C57Bl/6J mouse reference sequence (GenBank Accession number NC_005089) and the consensus DNA sequence for that mouse using SeqScape software (Applied Biosystems) to determine the somatic mtDNA point mutations that accumulated in the crypts over time. This was repeated and PCR products resequenced to confirm the somatic mtDNA mutations detected. Heteroplasmy levels were estimated based upon the relative peak height of electropherograms.

### 2.4 Long-range PCR of individual crypts

The single crypt lysate (see above) was used as the DNA template for long-range PCR to determine whether large-scale circular mtDNA deletions were present. Two rounds of PCR were carried out. PCR was performed using Takara *LA*Tak PCR system (Takara Bio Inc.) according to manufacturer's recommended conditions. After 30 cycles of first round PCR (95 °C for 20 s, 68 °C for 16 min), 2 μl of the first round product was used as template for the second round PCR reaction (same as above) and 20 further cycles of PCR carried out. First round PCR primers were L272–L301 and H16286–H16254; second round primers were L1275–L13004 and H15833–H15804 giving an expected product size of ∼14.5 kb PCR products were subjected to electrophoresis through a 0.8% agarose gel and the band sized against a 1 kb Ladder (Promega).

### 2.5 In silico modelling of clonal expansion of mtDNA mutations

We developed a stochastic model of mtDNA mutation expansion in stem cells by random genetic drift. The model was designed and run in MATLAB (version 8.0 MathWorks, Massachusetts, United States) and is available from the author upon request (CS). Each stem cell contains a constant number of 200 mtDNA molecules (Coller et al., 2001), which replicate (relaxed replication) during each cell cycle and are then segregated symmetrically to the daughter cells. After the division one of the daughter cells is kept and the other discarded along with the mtDNA molecules it contains (asymmetric division). In each division there is a certain probability (equal to the mtDNA mutation rate) that a pathogenic mtDNA mutation occurs. The mutated mtDNA molecule can then clonally expand to take over the stem cell, which becomes COX deficient. To match the experimentally observed COX deficiency data from WT, *PolgA*^*+/mut*^ and *PolgA*^*mut/mut*^ mice, we performed a parameter scan of the mtDNA mutation rate, assuming >75% mutated mtDNA was enough to confer COX deficiency. The mtDNA mutation frequency was determined by averaging 1000 simulation runs of 1080 divisions (36 months). Full details are included in Supplementary File 5.

## 3 Results

### 3.1 Respiratory chain deficiency in colonic crypts

**Fig. 1:**
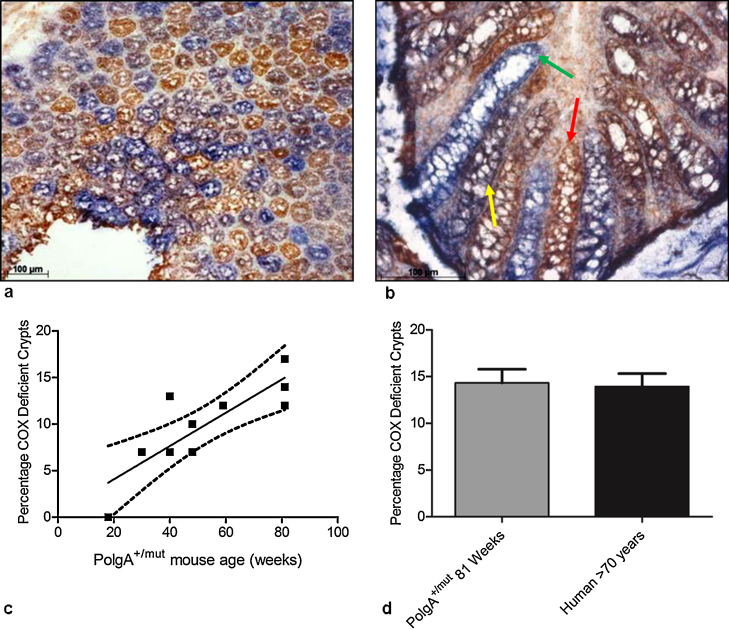
Respiratory chain deficiency in the ageing *PolgA^+/mut^* mouse colon. (a) COX/SDH histochemistry on 81 week old *PolgA^+/mut^* mouse colon, showing a transverse section through the crypts. (b) COX/SDH histochemistry on 81 week old *PolgA^+/mut^* mouse colon, showing a longitudinal section through the crypts. Scale bars: 100 μm. Crypts stained brown are positive for COX activity (red arrow), those stained blue are COX deficient (green arrow), and crypts stained purple/grey display intermediate COX deficiency (yellow arrow). Note that in the COX deficient crypt (green arrow) the COX deficiency extends throughout the crypt, including the stem cell compartment. (c) Incidence of COX deficient colonic crypts in *PolgA^+/mut^* mice aged 18–81 weeks old. Linear regression analysis, *p* = 0.0028. (d) Mean incidence (±SEM) of COX deficient colonic crypts in 81 week old *PolgA^+/mut^* mice and aged humans >70 years old (Taylor et al., 2003). The percentage of COX deficient colonic crypts was not significantly different between 81 week *PolgA^+/mut^* mice and aged humans >70 years old, *p* = 0.854 (unpaired *t* test).

Dual COX/SDH histochemistry and Haematoxylin and Eosin (H&E) histology were performed on colonic epithelial tissue of a series of ageing (18–81 weeks) *PolgA*^*+/mut*^ mice. The H&E staining showed normal colonic epithelial morphology. The COX/SDH histochemistry detected colonic crypts that were both partially and fully COX-deficient, present in a random, mosaic pattern throughout the tissue (Fig. 1a and b). The number of fully COX deficient colonic crypts increased markedly with age, from 0% in 18 weeks to ∼14% in 81 week old animals (Fig. 1c). The frequency of partially COX deficient crypts also increased with age from 0% in the youngest animals to 8% in the oldest. Analysis of longitudinal sections through COX deficient crypts (Fig. 1b) confirmed that, as in the human colon, COX deficiency was present from the base to the apex of the crypts, including within the stem cell compartment. When the data from the 81 week *PolgA*^*+/mut*^ mice were compared to the frequency of COX deficiency in the ageing human colon (Taylor et al., 2003), there was no significant difference (*p* = 0.854, unpaired *t* test) (Fig. 1d).

### 3.2 MtDNA mutations in *PolgA*^*+/mut*^ mouse colonic crypts

To determine whether COX deficient colonic crypts in the *PolgA*^*+/mut*^ mouse were due to clonally expanded mtDNA point mutations, we laser micro-dissected at least 8 individual COX deficient and 4 COX positive colonic crypts from three 81 week old *PolgA*^*+/mut*^ animals, and sequenced the entire mitochondrial genome. Long-range PCR was carried out to look for the presence of large-scale mtDNA deletions.

**Fig. 2:**
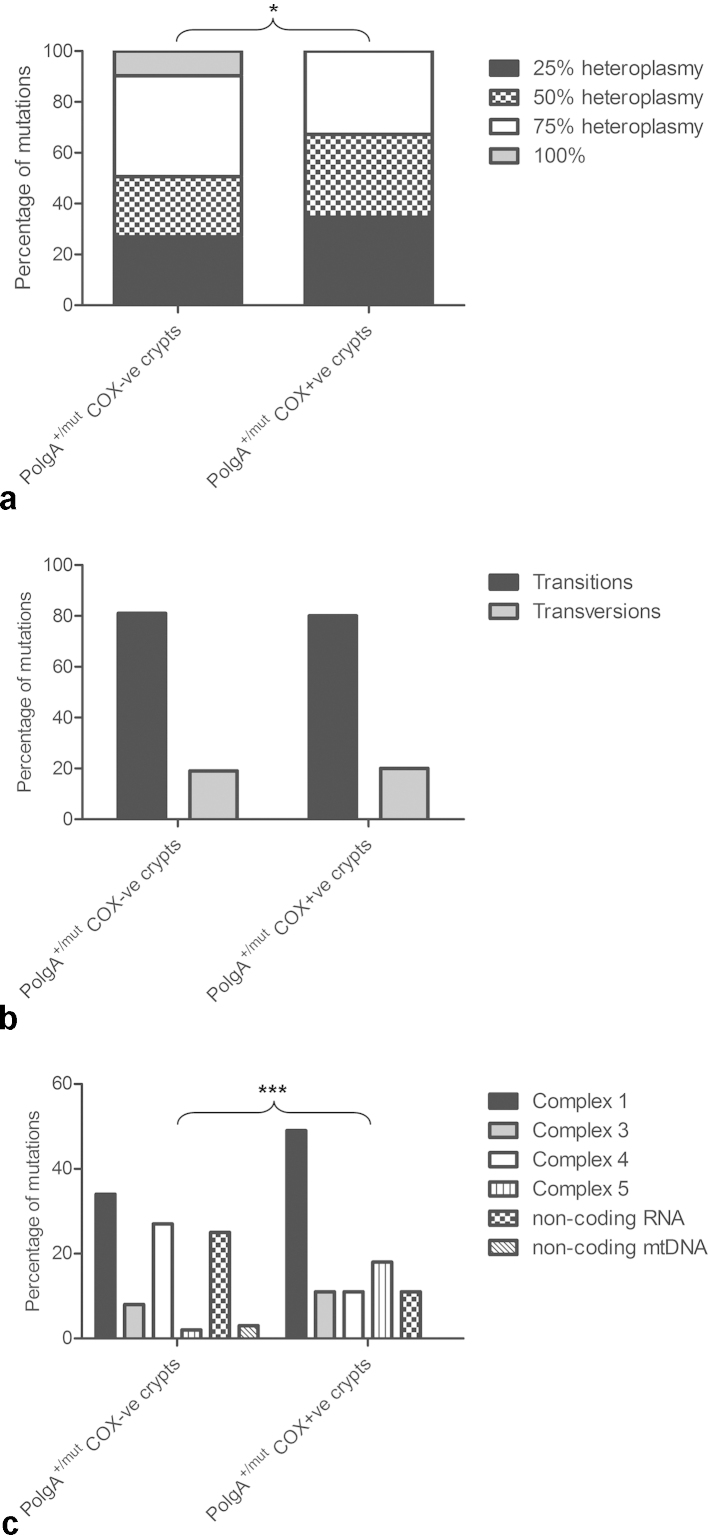
Frequency, type and location of somatic mtDNA point mutations in *PolgA^+/mut^* mouse colonic crypts. (a) The frequency of mutations across the different heteroplasmy classes in *PolgA^+/mut^* mouse COX deficient and COX positive colonic crypts. There was a significantly high frequency of mtDNA mutations in the higher heteroplasmy classes in the COX deficient crypts (*χ*^2^ analysis *p* = 0.0439). (b) Types of changes observed in COX deficient and COX positive colonic crypts. There was no significant difference in the types of changes between the two (*χ*^2^ test *p* = 1.000). (c) Gene location of mutations in individual mtDNA encoded genes in COX deficient and positive colonic crypts. There was a significant difference between the location of the mtDNA mutations detected in COX positive and COX deficient crypts (*χ*^2^ test *p* = <0.0001).

No large-scale mtDNA deletions were detected in either COX-positive or COX deficient crypts (Supplementary File 1a). In contrast, somatic mtDNA point mutations were detected in both COX-positive and COX deficient *PolgA*^*+/mut*^ mouse colonic crypts, (Supplementary File 2). In the COX positive crypts the majority (67%) of the mtDNA point mutations detected were present at low levels of heteroplasmy (≤50%) and were therefore unlikely to affect mitochondrial function (Fig. 2a). Eighty percent of the changes observed were base transitions (Fig. 2b) and 49% affected complex I genes (Fig. 2c). In COX deficient crypts, 81% of the mutations detected were base transitions, predominantly C > T transitions (49%) and G > A changes (17%) (Fig. 2b). They were mainly present in protein encoding genes with 34% in complex I genes and 27% in complex IV genes (Fig. 2c). Seventy-three percent of these mutations were present at heteroplasmy levels ≥50% and were non-synonymous mutations predicted to cause amino acid substitutions (Fig. 2a and Supplementary File 2). Twenty-five percent of the mutations were detected in mt-tRNA and mt-rRNA genes at heteroplasmy levels of ≥75% up to homoplasmy. Example electropherograms of homoplasmic and heteroplasmic mutations detected in COX deficient crypts are shown in Supplementary File 1b.

**Fig. 3:**
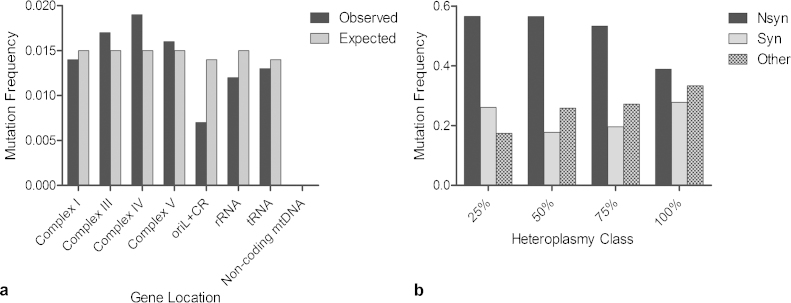
MtDNA mutations occur randomly in *PolgA^+/mut^* mouse colonic crypts. (a) Positional mutation frequency of observed vs expected (number of mutations/base pairs) somatic mtDNA mutations in the different gene types detected in *PolgA^+/mut^* colonic crypts. There was no significant difference between the observed and expected frequencies, (*p* = 0.139, *χ*^2^ test). Abbreviations: oriL, origin of light strand replication; CR, control region. (b) The mutation frequency of non-synonymous, synonymous and “other” changes across the different heteroplasmy classes in the *PolgA^+/mut^* mouse colonic crypts. There was no significant difference in the types of mutations observed between the heteroplasmy classes (*χ*^2^ analysis, *p* = 0.6765) Abbreviations: Nsyn, non-synonymous and syn, synonymous changes.

To investigate whether somatic mtDNA mutations occurred at random in the *PolgA*^*+/mut*^ mouse colon we grouped the mutations according to gene location. There was no significant difference between the observed frequencies of somatic mutations in each gene type and those expected due to random chance, based on the relative proportion of the genome occupied by each gene (*p* = 0.139, *χ*^2^ test, Fig. 3a). Furthermore, there was no significant difference in the ratio of synonymous: non-synonymous changes across the different heteroplasmy classes (*p* = 0.6765, *χ*^2^ test, Fig. 3b), suggesting a lack of mutational bias towards the clonal expansion of deleterious mutations in the *PolgA*^*+/mut*^ colon.

### 3.3 The levels of clonally expanded somatic point mutations of mtDNA are similar in the colon from ageing *PolgA*^*+/mut*^ mice and humans

**Fig. 4:**
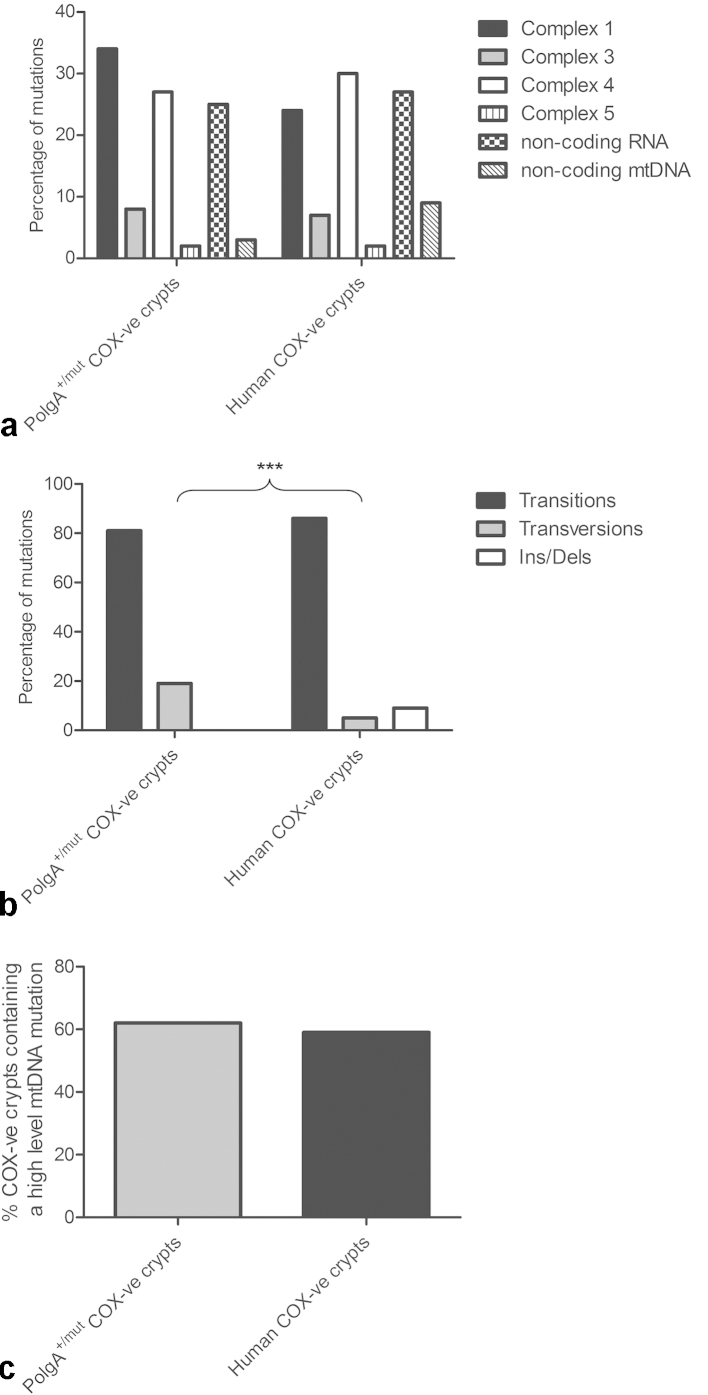
Gene type, location and clonal expansion of somatic mtDNA point mutations in the ageing *PolgA^+/mut^* mouse and human colon. (a) Gene location of mutations in individual mtDNA encoded genes in COX deficient colonic crypts. There was no significant difference between the *PolgA^+/mut^* mouse crypts and the human crypts (*χ*^2^ test *p* = 0.2204). (b) Types of changes observed in COX deficient colonic crypts. There was a significantly higher frequency of insertion/deletion mutations in the human crypts (*χ*^2^ test *p* = <0.001). (c) Percentage of COX deficient colonic crypts containing at least one pathogenic mtDNA point mutation in the *PolgA^+/mut^* mouse and human colon. There was no significant difference between the mouse and human crypts (*χ*^2^ test *p* = 0.8194).

A comparison of the nature of somatic mtDNA mutations causing COX deficiency in colonic crypts of *PolgA*^*+/mut*^ mice and ageing humans was performed (Greaves et al., 2010, Greaves et al., 2012, Greaves et al., 2006, Taylor et al., 2003) (Supplementary File 3). There was no significant difference in the gene location of the mutations in COX deficient crypts in the *PolgA*^*+/mut*^ mouse and human colon, with 60% of the mutations affecting genes encoding subunits of complexes I and IV, and 25% in non-coding RNA (tRNA and rRNA) genes in both datasets (*p* = 0.2204, *χ*^2^ test, Fig. 4a).

Next we compared the types of mtDNA mutations (transitions, transversions and insertions/deletions) and found a significant difference in the types of mutations detected in COX deficient colonic crypts between *PolgA*^*+/mut*^ mice and humans (*p* = <0.0001, *χ*^2^ test, Fig. 4b). Although the majority of changes were base transitions (∼80%) in both data sets, no insertions/deletions were detected in *PolgA*^*+/mut*^ mouse colonic crypts, whereas they accounted for ∼10% of changes in the ageing human colon (Greaves et al., 2012). Additionally, a higher proportion of transversions were observed in COX deficient crypts from the *PolgA*^*+/mut*^ mice (19%) than in humans (5%).

To determine whether the mechanism underlying COX deficiency in colonic crypts was the same in the human and *PolgA*^*+/mut*^ mouse data sets, somatic mtDNA mutations were assigned pathogenicity according to the following criteria: (1) not previously identified as a polymorphic variant (as reported in Genbank or transmitted through the *PolgA*^*mut/mut*^ germline (Stewart et al., 2008b)); (2) changed an amino acid or occurred in a tRNA or rRNA gene; (3) present at levels >50%; and (4) associated with decreased activity of a respiratory chain complex (COX deficiency) (Greaves et al., 2010, Taylor et al., 2003). The frequency of pathogenic mtDNA point mutations was similar (*p* = 0.8194, *χ*^2^ test) in COX deficient colonic crypts from *PolgA*^*+/mut*^ mice (62%) and humans (59%) (Fig. 4c). This indicates that COX deficiency is caused by the clonal expansion of at least one pathogenic mtDNA point mutation in ∼60% of colonic crypts in both ageing humans and *PolgA*^*+/mut*^ mice.

### 3.4 No evidence for purifying selection of somatic mtDNA point mutations in colonic crypts from *PolgA*^*+/mut*^ mice

**Fig. 5:**
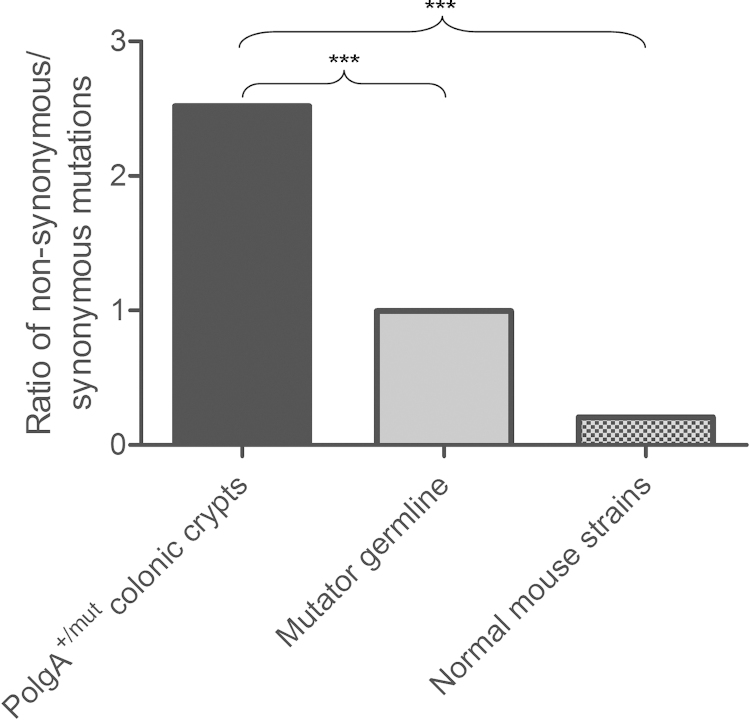
Genetic consequences of somatic mtDNA mutations in the *PolgA^+/mut^* mouse colon. The ratio of non-synonymous mutations: synonymous mutations in *PolgA^+/mut^* mouse colonic crypts, the mtDNA mutator mouse germline and normal mouse strains. The ratio of non-synonymous to synonymous mutations is significantly higher in *PolgA^+/mut^* mouse colonic crypts compared to both the mtDNA mutator mouse germline and the normal mouse strains (*p* = <0.0001, Fisher's exact test).

**Fig. 6:**
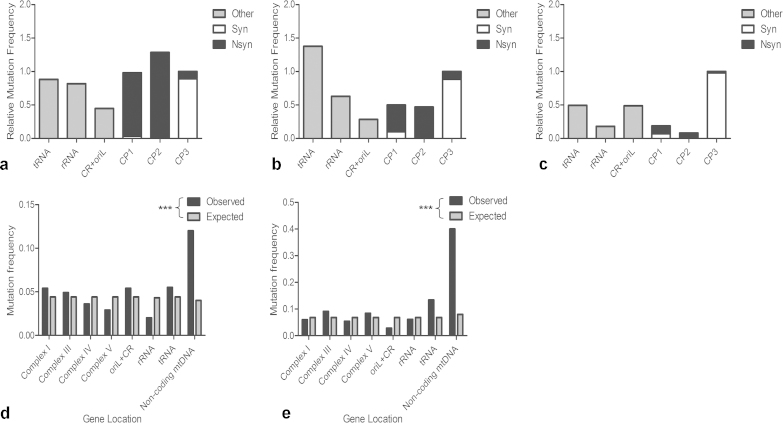
Positional mutation frequency and mutation distribution by codon position and gene reveals an absence of evidence for purifying selection on somatic mtDNA point mutations in *PolgA^+/mut^* colonic crypts. (a) Positional mutation frequency (observed mutations per base pair) of somatic mtDNA mutations observed in *PolgA^+/mut^* colonic crypts compared to (b) mtDNA mutations transmitted through the mtDNA mutator mouse germline (Stewart et al., 2008b) and (c) mtDNA sequences of mouse strains from Genback, *Mus musculus*. To compare between the classes we took the sum of the 3rd codon position and standardised that as 1 (value/3rd codon sum). CP1-3: codon positions 1–3. (d) Positional mutation frequency (number of mutations/base pairs) of observed vs expected (based on a random distribution) mtDNA point mutations transmitted through the mtDNA mutator mouse germline (Stewart et al., 2008b) (*p* = <0.0001, *χ*^2^ test) and (e) mtDNA sequences of mouse strains from Genbank, *Mus musculus* (*p* = <0.0001, *χ*^2^ test).

To determine whether somatic mtDNA mutations are subject to selective constraints in the ageing *PolgA*^*+/mut*^ mouse colon, we compared our dataset to *Mus musculus* mtDNA mutations reported in Genbank and to mtDNA mutations transmitted through the germline of mtDNA mutator mice (Stewart et al., 2008b). In the *PolgA*^*+/mut*^ mouse colonic crypts, the ratio of non-synonymous substitutions per non-synonymous site (dN) to synonymous substitutions per synonymous site (dS) gave a value of 0.783, providing no significant evidence of purifying selection against mutations causing amino acid substitutions in protein encoding genes (*p* = 0.3402, Fisher's exact test). dN/dS values of ∼1 signify an absence of selection on the analysed sequences. This was significantly different to mutations observed in the mtDNA mutator mouse germline (dN/dS = 0.310) and normal mouse strains (dN/dS = 0.0640) where mutations were more frequently synonymous changes (Fisher's exact test, *p* = <0.0001 in both cases) (Fig. 5). There was also a substantially higher number of mutations in the first and second codon positions of protein encoding genes of *PolgA*^*+/mut*^ mouse colonic crypts (Fig. 6a) compared to the mtDNA mutator mouse germline (Fig. 6b) and normal mouse strains (Fig. 6c), further demonstrating an absence of evidence for purifying selection, as changes in the first and second codon positions frequently result in amino acid substitution (Stewart et al., 2008a, Stewart et al., 2008b). Furthermore, the random distribution of mtDNA point mutations observed in the *PolgA*^*+/mut*^ mouse colonic crypts (Fig. 3a) was not observed in either the mtDNA mutator mouse germline (Fig. 6d) or normal mouse strains (Fig. 6e), which both show a non-random distribution of mtDNA mutations, with purifying selection against protein encoding mutations (*p* = 0.0005 and *p* = <0.0001 in mutator mouse germline and normal mouse strains respectively, *χ*^2^ test).

### 3.5 Random genetic drift can explain the expansion of mtDNA mutations in the colonic crypts of both *PolgA*^*+/mut*^ mice and humans

Random genetic drift has been hypothesised to be a plausible explanation for the clonal expansion of mtDNA mutations in both mitotic (Coller et al., 2001, Taylor et al., 2003) and post-mitotic human cells (Elson et al., 2001), leading to respiratory chain deficiency. There are questions as to whether this could also be a plausible mechanism of clonal expansion in short-lived animals, as modelling studies have shown that in post-mitotic tissues such a mechanism cannot explain the experimental data (Kowald and Kirkwood, 2013), however such modelling has not been carried out in mitotic tissues from short-lived animals where it could be argued that many cell generations may elapse even within a short lifespan.

**Fig. 7:**
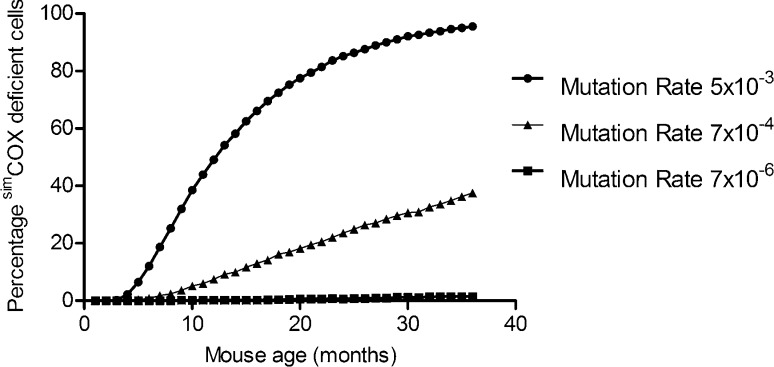
In silico modelling of the clonal expansion of mitochondrial DNA mutations in colonic crypt stem cells. Each symbol is the mean of 1000 simulated cells, each containing 200 mtDNA molecules with the mutation rates described, with a cell division rate of once per day for 3 years. To match the experimentally observed COX deficiency data from WT (Greaves et al., 2011), *PolgA^+/mut^* (Fig. 1) and *PolgA^mut/mut^* mice (Supplementary File 4), we performed a parameter scan of the mtDNA mutation rate, assuming >75% mutated mtDNA was enough to confer COX deficiency. The mtDNA mutation frequency was determined by averaging of 1000 simulation runs of 1080 divisions (36 months). The proportion of simulated cells containing >75% mutant mtDNA (^sim^COX_deficient_ cells) predicted by the model at the three mutation rates shown, closely matches our experimental data from wild-type, *PolgA^+/mut^*, and *PolgA^mut/mut^* mice.

To determine whether the frequency of COX deficient crypts observed in the *PolgA*^*+/mut*^ mice compared to wild-type was the result of accelerated clonal expansion by random genetic drift, a computational simulation was developed based on previously established models and experimental data (Coller et al., 2001, Elson et al., 2001, Taylor et al., 2003, Vermulst et al., 2007). The effects of increasing mtDNA mutation rate on frequency of COX deficient crypts in the *PolgA*^*+/mut*^, and wild-type mice were simulated. For completeness, the frequency of COX deficient crypts in 3, 6, 9 and 12 month old *PolgA*^*mut/mut*^ mice was calculated and these data were included in the simulation (Supplementary File 4). All simulation parameters are detailed in Supplementary File 5. The results of the simulations show that changing only the mtDNA mutation rate can lead to accelerated clonal expansion and the levels of COX deficiency we observe experimentally (Fig. 7). This suggests that, like in the human system, random genetic drift can explain the clonal expansion of mtDNA mutations in the colonic crypts of mice during ageing.

## 4 Discussion

Here we have demonstrated that *PolgA*^*+/mut*^ mice display evidence of age-related clonally expanded somatic mtDNA point mutations in the colonic epithelium, similar to the findings in normal human ageing. We have shown that *PolgA*^*+/mut*^ mice accumulate COX deficient colonic crypts with age and by 81 weeks of age their phenotype is very similar to the pattern of COX deficient colonic crypts found in 70-year-old human subjects (Taylor et al., 2003). We have identified that COX deficiency is associated with the clonal expansion of random, somatic mtDNA point mutations, and that there is no evidence of purifying selection in this tissue, similar to observations in ageing humans (Greaves et al., 2012). Furthermore, we have demonstrated that the random genetic drift model of clonal expansion, which has been shown to model clonal expansion in human colonic epithelium very well (Taylor et al., 2003), can also model the mitochondrial phenotypes observed in wild-type and *PolgA*^*+/mut*^ mice.

We did observe differences in the types of mtDNA point mutations detected in *PolgA*^*+/mut*^ mouse and human COX deficient colonic crypts, principally in terms of frameshift mutations. This was not surprising given the fact that the *PolgA*^*+/mut*^ mice are engineered to have a proof-reading deficient polymerase and in a recent study involving mtDNA mutator mice <1% of the mutations were insertions/deletions in a dataset of 442 mutations (Ross et al., 2013). Thus the moderate size of our mtDNA mutation dataset (241 mutations) and the relatively low insertion/deletion rate in *PolgA*^*+/mut*^ mice renders it unlikely that we would observe any insertions/deletions. Nevertheless, the majority of the mutations in both the *PolgA*^*+/mut*^ mouse and human colon were base transitions, supporting the hypothesis that somatic mtDNA point mutations occur early in life due to mtDNA replication errors and/or spontaneous cytosine deamination (Vermulst et al., 2007) and clonally expand throughout adulthood (Coller et al., 2001, Elson et al., 2001). Only a minor number of base transversions, which were principally A-T changes, were observed in both humans and mice providing little evidence of 8-oxo-deoxyguanine mediated mutagenesis and oxidative damage.

This study confirms that mutations in genes other than *MT-CO* genes can result in COX deficiency, most commonly mutations in non-coding RNA genes (tRNAs and rRNAs), which can cause translational defects, affecting the abundance and activity of mtDNA encoded respiratory chain subunits. Interestingly, in both the *PolgA*^*+/mut*^ mice and ageing humans, we also noted that some COX deficient crypts contained only one pathogenic mutation in a complex I gene. Respiratory chain complexes are believed to associate to form supercomplexes (Schagger and Pfeiffer, 2000) and in patients with mitochondrial disease, mutations in structural subunits of complex I have been associated with multiple respiratory chain defects (Hinttala et al., 2006). Furthermore, complex I defects are frequently associated with complex IV defects in the ageing human colon (Greaves et al., 2010). Thus it is possible that a single pathogenic complex I mutation may also affect the stability and activity of complex IV and play a causal role in the COX deficiency. We observed colonic crypts that did not contain a single pathogenic mtDNA point mutation. However, these crypts commonly presented with multiple heteroplasmic mutations and the observed COX deficiency may therefore have been caused by a combined effect of multiple heteroplasmic mutations.

In the *PolgA*^*mut/mut*^ mouse germline there is strong purifying selection against mtDNA mutations in the first and second codon positions of protein encoding genes, as well as a reduction in tRNA and rRNA mutations (Stewart et al., 2008b). This was significantly different to what we observed in the *PolgA*^*+/mut*^ mouse colon, confirming that mtDNA in germ cells and mtDNA in ageing somatic tissues appear to be subject to different selection pressures (Greaves et al., 2012). These differences are likely due to selection for mitochondrial fitness during oocyte development via the mtDNA bottleneck to protect the germline from deleterious mtDNA mutations (Stewart et al., 2008a), and a subsequent lack of selection in the somatic tissues, agreeing with the disposable soma theory of ageing (Kirkwood, 1977). Despite protective mechanisms in the germline, low level heteroplasmic mtDNA mutations are still inherited (He et al., 2010, Payne et al., 2013) and pre-existing germline mtDNA point mutations have been shown to accelerate the clonal expansion of somatic mtDNA point mutations in ageing (Ross et al., 2013). Our data lends further support to this as increased levels of respiratory chain deficiency and somatic mtDNA mutations are observed in the colon of *PolgA*^*+/mut*^ mice, which have a higher germline mutation frequency than normal ageing mice (Greaves et al., 2011).

Our model of mtDNA point mutation clonal expansion by random genetic drift has shown that a simple increase in the mtDNA mutation rate can explain the differences in the frequencies of COX deficient crypts we see between the wild-type and *PolgA*^*+/mut*^ mice. These modelling simulations are in contrast to those carried out previously which concentrated on post-mitotic tissues from short-lived animals and showed that random genetic drift is unable to explain the experimental data on incidence of COX deficient cells in rats (Kowald and Kirkwood, 2013). These apparently conflicting data may be attributable to tissue-specific differences. Data from a study using heteroplasmic mice containing two different mtDNA genotypes in which the authors measured the relative contribution of each genotype in individual colonic crypts over time showed that in individual crypts from 4 month old mice there was a mixture of the two genotypes, however in the crypts of the 15 month old mice two distinct crypt populations were observed with one or the other genotypes predominating. These data fit with a model of neutral drift (Jenuth et al., 1997), however when they looked at liver, kidney, spleen and blood, the genotypes segregated in a very different manner, with strong tissue-specific selection for one genotype or the other. In addition, in post-mitotic tissues in these mice, there was no segregation of the mutations at all, with similar proportions of each genotype present throughout the life-course (Jenuth et al., 1997). Thus, whilst our data from the colon are compatible with random genetic drift as a mechanism for clonal expansion of mtDNA point mutations in both mice and humans; this may not be applicable to all tissues.

## 5 Conclusions

Here we show that clonal expansion of somatic mtDNA mutations without clear selective constraints leads to respiratory chain deficiency in the ageing of colonic epithelial tissue both in *PolgA*^*+/mut*^ mice and humans. This indicates that the *PolgA*^*+/mut*^ mouse may be a valuable model in which to study the cellular responses invoked by the mosaic tissue distribution of mitochondrial dysfunction that occurs with age. Given the fact that stem cell markers are much more well-characterised in mice (Barker et al., 2007, Itzkovitz et al., 2012), we believe that the *PolgA*^*+/mut*^ mouse could provide information that is currently lacking about the effects of clonally expanded mtDNA mutations on human stem cell function and normal tissue homeostasis, in both ageing and age-related disease.

## Funding

HLB, LCG, and DMT were supported by the Newcastle University Centre for Brain Ageing and Vitality supported by BBSRC, EPSRC, ESRC and MRC as part of the cross-council Lifelong Health and Wellbeing Initiative (G0700718). CS and AZ were supported by the 10.13039/501100000268BBSRC (BB/F016980/1 and BB/H011471/1). TBLK was supported by the National Institute for Health Research Newcastle Biomedical Research Centre (17/08/2011) based at Newcastle Hospitals NHS Foundation Trust and Newcastle University. The views expressed are those of the author(s) and not necessarily those of the NHS, the NIHR or the Department of Health. DMT was also supported by the 10.13039/100004440Wellcome Trust (Strategic Award 096919/Z/11/Z).

## Author contributions

HLB and JBS carried out the experimental procedures; CS, AZ and TBLK carried out the in silico modelling; JBS carried out statistical analysis; LCG, DMT and NGL designed the study; HLB and LCG wrote the paper. All authors commented on and made intellectual contributions to the manuscript at all stages of writing.

## Appendix A Supplementary data

Supplementary File 1 Mutational analysis of colonic crypts from *PolgA*^*+/Mut*^ mice.

Supplementary File 2 Mitochondrial DNA mutations detected in individual colonic crypts from *PolgA*^*+/mut*^ mice.

Supplementary File 3 Table comparing the location, types and frequency of mtDNA point mutations in colonic crypts from *PolgA*^*+/Mut*^ mice and aged humans.

Supplementary File 4 Respiratory chain deficiency in ageing *PolgA*^*mut/mut*^ mouse colon.

Supplementary File 5 Description of in silico modelling of clonal expansion of mtDNA mutations.
