# Supplementary material for: Similar patterns of clonally expanded somatic mtDNA mutations in the colon of heterozygous mtDNA mutator mice and ageing humans
Source: Mech Ageing Dev. 2014 Jul;139:22–30. doi: 10.1016/j.mad.2014.06.003 (PMC4141908; doi:10.1016/j.mad.2014.06.003)
Supplement: Supplementary File 1 — Mutational analysis of colonic crypts from PolgA+/Mut mice. [file mmc1.pdf]

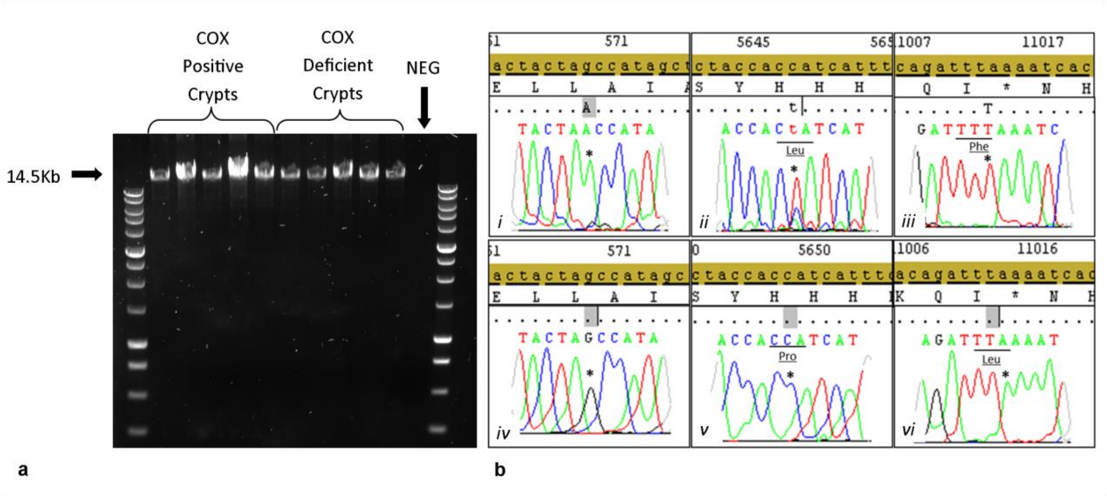

Supplementary File 1: Mutational analysis of crypts from *PolgA*$^{+/Mut}$ mice. (**a**): Example 0.8% Agarose gel showing Long-range PCR products. The wild-type band is 14.5Kb; deleted species would appear as smaller bands. Only full-length bands were detected in both COX-positive and COX deficient crypts. (**b**) Sequencing electropherograms showing examples of point mutations detected in COX deficient colonic crypts from *PolgA*$^{+/mut}$ mice. (***i***) Electropherogram showing the homoplasmic m. 568 G>A transition in *MT-srRNA*. (**ii**) Electropherogram showing the heteroplasmic (75%) m.5647 C>T transition. This change predicts a proline to leucine amino acid change in *MT-CO1*. (**iii**) Electropherogram showing the homoplasmic m.11012 A>T transversion. This change predicts a leucine to phenylalanine amino acid change in *MT-ND4*. Wild-type homogenate sequences are shown in panels (**iv-vi**).
