# Supplementary material for: Similar patterns of clonally expanded somatic mtDNA mutations in the colon of heterozygous mtDNA mutator mice and ageing humans
Source: Mech Ageing Dev. 2014 Jul;139:22–30. doi: 10.1016/j.mad.2014.06.003 (PMC4141908; doi:10.1016/j.mad.2014.06.003)
Supplement: Supplementary File 2 — Mitochondrial DNA mutations detected in individual colonic crypts from PolgA+/mut mice. [file mmc2.pdf]

Crypt	COX Histochemistry	Mutation	WT base	Mutant base	Heteroplasmy level	Gene	Codon change	Amino acid change	Substitution type
1	Deficient	m.4892 T>C	T	C	100%	*MT-ND2*	TTC	Phe-Phe	syn
	Deficient	m.6779 T>C	T	C	25%	*MT-CO1*	CCA	Ser-Pro	ns
2	Deficient	m.4892 T>C	T	C	100%	*MT-ND2*	TTC	Phe-Phe	syn
	Deficient	m.5831 T>C	T	C	50%	*MT-CO1*	ACT	Ile-Thr	ns
	Deficient	m.7413 C>T	C	T	50%	*MT-CO2*	TGA	Arg-Trp	ns
	Deficient	m.9740 T>C	T	C	100%	*MT-ND3*	CCA	Leu-Pro	ns
	Deficient	m.10392 A>G	A	G	25%	*MT-ND4*	CTG	Leu-Leu	syn
	Deficient	m.13817 G>T	G	T	25%	*MT-ND6*	AAA	Asn-Lys	ns
	Deficient	m.13852 C>T	C	T	50%	*MT-ND6*	ACT	Ala-Thr	ns
3	Deficient	m.6098 T>A	T	A	75%	*MT-CO1*	GAA	Val-Glu	ns
4	Deficient	m.986 G>A	G	A	50%	*MT-16SrRNA*	-	-	
	Deficient	m.2283 G>T	G	T	50%	*MT-16SrRNA*	-	-	
	Deficient	m.6024 G>A	G	A	75%	*MT-CO1*	CAA	Gln-Gln	syn
5	Deficient	m.1214 C>G	C	G	75%	*MT-16SrRNA*	-		
	Deficient	m.5695 G>A	G	A	100%	*MT-CO1*	AGA	Gly-Gly	syn
	Deficient	m.6835 C>T	C	T	75%	*MT-CO1*	TAC	His-Tyr	ns
	Deficient	m.6919 C>T	C	T	100%	*MT-tRNA* ^{Ser}	-		
	Deficient	m.7641 G>A	G	A	100%	*MT-CO2*	ATC	Val-Ile	ns
6	Deficient	m.506 C>T	C	T	75%	*MT-12SrRNA*	-	-	
	Deficient	m.812 C>G	C	G	75%	*MT-12SrRNA*	-	-	
	Deficient	m.2424 C>T	C	T	50%	*MT-16SrRNA*	-	-	
	Deficient	m.2446 C>T	C	T	75%	*MT-16ArRNA*	-	-	
	Deficient	m.4946 A>G	A	G	75%	*MT-ND2*	GCC	Thr-Ala	ns
	Deficient	m.11539 C>T	C	T	25%	*MT-ND4*	ATA	Thr-Met	ns
	Deficient	m.15243 C>T	C	T	50%	*MT-CYB*	TCA	Pro-Ser	ns
7	Deficient	m.3429 G>A	G	A	75%	*MT-ND1*	AAG	Glu-Lys	ns
	Deficient	m.5713 A>G	A	G	25%	*MT-CO1*	TGC	Tyr-Cys	ns
	Deficient	m.5894 T>A	T	A	75%	*MT-CO1*	CTA	Leu_leu	syn

Crypt	COX Histochemistry	Mutation	WT base	Mutant base	Heteroplasmy level	Gene	Codon change	Amino acid change	Substitution type
	Deficient	m.6444 G>C	G	C	50%	*MT-CO1*	CTA	Val-Leu	ns
	Deficient	m.14400 G>A	G	A	25%	*MT-CYB*	AGA	Gly-Null	ns
	Deficient	m.16254 G>A	G	A	50%	*ctrl region*	-	-	-
8	Deficient	m.847 T>A	T	A	50%	*MT-12SrRNA*	-	-	
	Deficient	m.2537 G>A	G	A	50%	*MT-16SrRNA*	-	-	
	Deficient	m.4143 A>T	A	T	25%	*MT-ND2*	ATC	Asn-Ile	ns
	Deficient	m.5959 C>T	C	T	50%	*MT-CO1*	ATA	Thr-Met	ns
	Deficient	m.8126 C>T	C	T	50%	*MT-ATP6*	ATA	Thr-Met	ns
	Deficient	m.8744 C>T	C	T	75%	*MT-CO3*	GGT	Gly-Gly	syn
9	Deficient	m.1181 A>G	A	G	25%	*MT-16SrRNA*	-		
	Deficient	m.6305 C>T	C	T	25%	*MT-CO1*	ACT	Thr-Thr	syn
	Deficient	m.11402 T>A	T	A	50%	*MT-ND4*	ATA	Ile-Met	ns
	Deficient	m.12015 G>A	G	A	25%	*MT-ND5*	ATA	Val-Met	ns
10	Deficient	m.221 C>T	C	T	75%	*MT-12SrRNA*			
	Deficient	m.2649 C>T	C	T	50%	*MT-16SrRNA*			
	Deficient	m.2807 C>T	C	T	75%	*MT-ND1*	TTT	Phe-Phe	syn
	Deficient	m.2970 G>A	G	A	75%	*MT-ND1*	ACA	Ala-Thr	ns
	Deficient	m.6402 T>C	T	C	75%	*MT-CO1*	CCC	Ser-Pro	ns
	Deficient	m.7533 C>T	C	T	50%	*MT-CO2*	GTC	Ala-Val	ns
	Deficient	m.10360 T>C	T	C	25%	*MT-ND4*	TCA	Leu-Ser	ns
	Deficient	m.15357 C>T	C	T	75%	*Mt-tRNA P*			
11	Deficient	m.4925 C>T	C	T	25%	*MT-ND2*	TCC	Pro-Ser	ns
	Deficient	m.5714 C>T	C	T	25%	*MT-CO1*	TAT	Tyr-Tyr	syn
	Deficient	m.5977 C>T	C	T	25%	*MT-CO1*	ATA	Thr-Met	ns
12	Deficient	m.212 G>A	G	A	25%	*MT-12SrRNA*			
	Deficient	m.366 C>T	C	T	75%	*MT-12SrRNA*			
	Deficient	m.2745 C>T	C	T	100%	*MT-tRNA L1*			
	Deficient	m.3053 G>A	G	A	75%	*MT-ND1*	GGA	Gly-Gly	syn

Crypt	COX Histochemistry	Mutation	WT base	Mutant base	Heteroplasmy level	Gene	Codon change	Amino acid change	Substitution type
	Deficient	m.4830 C>T	C	T	25%	*MT-ND2*	CTA	Pro-Leu	ns
	Deficient	m.6640 G>A	G	A	75%	*MT-CO1*	CAA	Arg-Gln	ns
	Deficient	m.6886 C>T	C	T	75%	*MT-tRNA S1*			
	Deficient	m.8489 C>T	C	T	75%	*MT-ATP6*	ATA	Thr-Met	ns
	Deficient	m.9268 G>A	G	A	75%	*MT-CO3*	CAA	Gln-STOP	ns
	Deficient	m.10165 C>T	C	T	25%	*MT-ND4 L*	TAA	Ala-Val	ns
	Deficient	m.11062 C>T	C	T	50%	*MT-ND4*	GTA	Ala-Val	ns
	Deficient	m.11643 C>T	C	T	75%	*MT- tRNA S2*			
	Deficient	m.12862 G>A	G	A	75%	*MT-ND5*	GAA	Val-Glu	ns
	Deficient	m.12946 G>A	G	A	75%	*MT-ND5*	TAC	Cys-Tyr	ns
	Deficient	m.13268 A>G	A	G	25%	*MT-ND5*	ATG	Met-Met	syn
	Deficient	m.14471 T>C	T	C	75%	*MT-CYB*	TTC	Phe-Phe	syn
13	Deficient	m.4966 C>T	C	T	75%	*MT-tRNA W*			
	Deficient	m.5440 G>A	G	A	75%	*MT-CO1*	CAA	Arg-Gln	ns
	Deficient	m.10387 C>T	C	T	25%	*MT-ND4*	CTA	Pro-Leu	ns
	Deficient	m.10420 A>G	A	G	50%	*MT-ND4*	AGA	Lys-Null	ns
	Deficient	m.14309 C>T	C	T	50%	*MT-CYB*	TAT	Tyr-Tyr	syn
	Deficient	m.14326 C>T	C	T	50%	*MT-CYB*	ATA	Thr-Met	ns
	Deficient	m.15632 C>T	C	T	25%	*Ctrl region*			
14	Deficient	m.239 C>T	C	T	25%	*MT-12SrRNA*			
	Deficient	m. 2461 G>T	G	T	50%	*MT-16SrRNA*			
	Deficient	m.3278 G>A	G	A	25%	*MT-ND1*	CTA	Leu-Leu	syn
	Deficient	m. 3649 T>A	T	A	50%	*MT-ND1*	TAA	Leu-STOP	ns
	Deficient	m.5580 C>T	C	T	50%	*MT-CO1*	TTA	Leu-Leu	syn
	Deficient	m.7486 C>T	C	T	25%	*MT-CO2*	GAT	Asp-Asp	syn
	Deficient	m.12241 C>T	C	T	25%	*MT-ND5*	GTA	Ala-Val	ns
	Deficient	m.12986 C>T	C	T	25%	*MT-ND5*	GCT	Ala-Ala	syn
	Deficient	m.12997 C>T	C	T	25%	*MT-ND5*	ATA	Thr-Met	ns

Crypt	COX Histochemistry	Mutation	WT base	Mutant base	Heteroplasmy level	Gene	Codon change	Amino acid change	Substitution type
	Deficient	m.14617 C>T	C	T	75%	*MT-CYB*	ATA	Thr-Met	ns
15	Deficient	m.9077 c>T	C	T	25%	*MT-CO3*	AAT	Asn-Asn	syn
	Deficient	m.14537 C>T	C	T	25%	*MT-CYB*	TAT	Tyr-Tyr	syn
	Deficient	m.15529 A>T	A	T	25%	*Ctrl Region / ETAS2*			
16	Deficient	m.491 G>T	G	T	50%	*MT-12SrRNA*			
	Deficient	m.501 C>T	C	T	25%	*MT-12SrRNA*			
	Deficient	m.1001 A>T	A	T	25%	*MT-12SrRNA*			
	Deficient	m.2411 C>T	C	T	25%	*MT-16SrRNA*			
	Deficient	m.6186 G>A	G	A	25%	*MT-CO1*	ATA	Val-Met	ns
	Deficient	m.6418 A>T	A	T	75%	*MT-CO1*	GTC	Asp-Val	ns
	Deficient	m.7772 C>T	C	T	50%	*MT-ATP8*	TAA	Gln-STOP	ns
	Deficient	m.8109 C>T	C	T	50%	*MT-ATP6*	CAT	His-His	syn
17	Deficient	m.5032 C>T	C	T	75%	*MT-tRNA A*			
	Deficient	m.9430 T>C	T	C	75%	*MT-tRNA G*			
	Deficient	m.9666 G>A	G	A	75%	*MT-ND3*	ACT	Ala-Thr	ns
	Deficient	m.11317 C>T	C	T	75%	*MT-ND4*	TTA	ser-Leu	ns
	Deficient	m.12218 C>T	C	T	75%	*MT-ND5*	TAT	Tyr-Tyr	syn
	Deficient	m.13403 C>T	C	T	75%	*MT-ND5*	GAT	Asp-Asp	syn
	Deficient	m.15005 A>T	A	T	25%	*MT-CYB*	AAT	Lys-Asn	ns
	Deficient	m.15298 G>A	G	A	75%	*MT-tRNA T*			
18	Deficient	m.5718 C>T	C	T	50%	*MT-CO1*	TCT	Pro-ser	ns
	Deficient	m.11508 A>T	A	T	50%	*MT-ND4*	TCT	Thr-Ser	ns
	Deficient	m.13783 C>T	C	T	75%	*MT-ND6*	GTA	Val-Val	syn
19	Deficient	m.5976 A>T	A	T	25%	*MT-CO1*	TCA	Thr-Ser	ns
20	Deficient	m.2083 G>A	G	A	75%	*MT-16SrRNA*			
	Deficient	m.2386 A>T	A	T	50%	*MT-16SrRNA*			
	Deficient	m.4879 C>T	C	T	25%	*MT-ND2*	CCT	Pro-Pro	syn

Crypt	COX Histochemistry	Mutation	WT base	Mutant base	Heteroplasmy level	Gene	Codon change	Amino acid change	Substitution type
	Deficient	m.5096 G>A	G	A	75%	*MT-tRNA N*			
	Deficient	m.5647 C>T	C	T	75%	*MT-CO1*	CTA	Pro-Leu	ns
	Deficient	m.6241 T>C	T	C	50%	*MT-CO1*	TCT	Phe-Ser	ns
	Deficient	m.6850 C>T	C	T	50%	*MT-CO1*	CTA	Pro-Leu	ns
	Deficient	m.8864 C>G	C	G	75%	*MT-CO3*	TTG	Phe-Leu	ns
	Deficient	m.9985 G>A	G	A	75%	*MT-ND4L*	ATA	Val-Met	ns
	Deficient	m.11710 C>T	C	T	75%	*MT- tRNA l2*			
	Deficient	m.13789 C>T	C	T	50%	*MT-ND6*	TTA	Leu-Leu	syn
	Deficient	m.13967 C>T	C	T	75%	*MT-ND6*	AAT	Ser-Asn	ns
21	Deficient	m.14808 C>T	C	T	100%	*MT-CYB*	TCC	Pro-Ser	ns
	Deficient	m.14818 C>T	C	T	25%	*MT-CYB*	ATA	Thr-Met	ns
	Deficient	m.14925 C>T	C	T	100%	*MT-CYB*	TCA	Pro-Ser	ns
	Deficient	m.14938 C>T	C	T	25%	*MT-CYB*	CTA	Pro-Leu	ns
22	Deficient	m.5536 T>A	T	A	75%	*MT-CO1*	GAA	Val-Glu	ns
	Deficient	m.6964 C>T	C	T	75%	*MT-tRNA D*	-		-
	Deficient	m.7164 C>T	C	T	75%	*MT-CO2*	ATA	Thr-met	ns
	Deficient	m.9520 C>T	C	T	75%	*MT-ND3*	GTA	Ala-Val	ns
	Deficient	m.16166 A>T	A	T	75%	*ctrl region*	-		-
	Deficient	m.16286 A>G	A	G	75%	*ctrl region*	-		-
23	Deficient	m.2679 A>T	A	T	100%	*MT-tRNA L1*	-		-
	Deficient	m.4890 T>A	T	A	50%	*MT-ND1*	TAT	Phe-Tyr	ns
	Deficient	m.5686 C>T	C	T	25%	*MT-CO1*	GTA	Ala-Val	ns
	Deficient	m.6342 C>T	C	T	75%	*MT-CO1*	TTA	Leu-Leu	syn
	Deficient	m.11012 A>T	A	T	100%	*MT-ND4*	TTT	Leu-Phe	ns
	Deficient	m.11429 C>T	C	T	25%	*MT-ND4*	AAT	Asn-Asn	syn
	Deficient	m.12080 C>T	C	T	100%	*MT-ND5*	AAT	Asn-Asn	syn
	Deficient	m.12143 C>T	C	T	100%	*MT-ND5*	GCT	Ala-Ala	syn
24	Deficient	m.2078 A>T	A	T	100%	*MT-16SrRNA*			

Crypt	COX Histochemistry	Mutation	WT base	Mutant base	Heteroplasmy level	Gene	Codon change	Amino acid change	Substitution type
	Deficient	m.4885 A>T	A	T	75%	*MT-ND2*	CTT	Leu-Leu	syn
	Deficient	m.6472 C>T	C	T	75%	*MT-CO1*	TTA	ser-Leu	ns
	Deficient	m.9203 G>A	G	A	75%	*MT-CO3*	ATA	Met-Met	syn
	Deficient	m.11951 C>T	C	T	100%	*MT-ND5*	ACT	Thr-Thr	syn
	Deficient	m.14905 A>G	A	G	75%	*MT-CYB*	GGC	Asp-Gln	ns
	Deficient	m.15921 T>A	T	A	100%	*Ctrl Region*			
25	Deficient	m.1776 T>A	T	A	75%	*MT-16SrRNA*	-		-
	Deficient	m.5521 T>C	T	C	50%	*MT-CO1*	ACA	Met-Thr	ns
	Deficient	m.5931 C>A	C	A	75%	*MT-CO1*	ATA	leu-Met	ns
	Deficient	m.6484 T>A	T	A	75%	*MT-CO1*	GAG	Val-Glu	ns
	Deficient	m.8627 A>G	A	G	50%	*MT-CO3*	GCG	Ala-Ala	syn
	Deficient	m.8631 C>T	C	T	50%	*MT-CO3*	TAC	His-Tyr	ns
	Deficient	m.8650 C>T	C	T	50%	*MT-CO3*	CTA	Pro-Leu	ns
	Deficient	m.9505 A>G	A	G	50%	*MT-ND3*	AGG	Thr-Null	ns
	Deficient	m.11172 C>T	C	T	75%	*MT-ND4*	TGG	Arg-Trp	ns
	Deficient	m.11698 G>A	G	A	25%	*MT-tRNA L2*	-		-
	Deficient	m.12218 C>T	C	T	50%	*MT-ND5*	TAT	Tyr-Tyr	syn
	Deficient	m.12706 C>T	C	T	25%	*MT-ND5*	CTA	Pro-Leu	ns
	Deficient	m.14462 A>T	A	T	50%	*MT-CYB*	TCT	Ser-Ser	syn
26	Deficient	m.192 A>G	A	G	50%	*MT-12SrRNA*	-		-
	Deficient	m.424 G>T	G	T	25%	*MT-12SrRNA*	-		-
	Deficient	m.3497 C>T	C	T	50%	*MT-ND1*	ATT	Ile-Ile	syn
	Deficient	m.3553 C>T	C	T	50%	*MT-ND1*	TTA	Ser-Leu	ns
	Deficient	m.3609 C>T	C	T	25%	*MT-ND1*	TAT	His-Tyr	ns
	Deficient	m.5697 A>G	A	G	75%	*MT-CO1*	GCA	Thr-Ala	ns
	Deficient	m.6047 C>T	C	T	75%	*MT-CO1*	CAT	His-His	syn
	Deficient	m.6386 A>G	A	G	75%	*MT-CO1*	CTG	Leu-Leu	syn
	Deficient	m.6444 G>A	G	A	75%	*MT-CO1*	ATA	Val-Met	ns

Crypt	COX Histochemistry	Mutation	WT base	Mutant base	Heteroplasmy level	Gene	Codon change	Amino acid change	Substitution type
	Deficient	m. 15323 A>G	A	G	25%	*MT-tRNA T*	-		-
27	Deficient	m.13033 C>T	C	T	25%	*MT-ND5*	ATA	Thr-Met	ns
	Deficient	m.14024 C>T	C	T	25%	*MT-ND6*	AAA	Cys-Lys	ns
28	Deficient	m.4879 C>T	C	T	25%	*MT-ND2*	CCT	Pro-Pro	syn
	Deficient	m.5096 G>A	G	A	50%	*MT-tRNA N*			
	Deficient	m.13457 C>T	C	T	25%	*MT-ND5*	AAT	Asn-Asn	syn
29	Deficient	m.568 G>A	G	A	100%	*MT-12SrRNA*			
	Deficient	m.635 C>T	C	T	75%	*MT-12SrRNA*			
	Deficient	m.655 C>T	C	T	75%	*MT-12SrRNA*			
	Deficient	m.2357 G>A	G	A	75%	*MT-16SrRNA*			
	Deficient	m.2712 A>T	A	T	75%	*MT-tRNA L1*			
	Deficient	m.2898 G>A	G	A	75%	*MT-ND1*	ACA	Ala-Thr	ns
	Deficient	m.5357 C>T	C	T	100%	*MT-CO1*	ACT	Thr-Thr	syn
	Deficient	m.6147 G>A	G	A	75%	*MT-CO1*	ATA	Val-Met	ns
	Deficient	m.6486 T>A	T	A	75%	*MT-CO1*	ATT	Phe-Ile	ns
	Deficient	m.10449 T>C	T	C	75%	*MT-ND4*	CAC	Tyr-His	ns
	Deficient	m.11236 C>T	C	T	75%	*MT-ND4*	ATA	Thr-Met	ns
	Deficient	m.12829 C>T	C	T	25%	*MT-ND5*	ATA	Thr-Met	ns
	Deficient	m.13971 C>T	C	T	75%	*MT-ND6*	TAA	Val-Leu	ns
1	Positive	m.3716 C > G	C	G	50%	*MT-tRNA I*	disrupts DHU stem		-
	Positive	m.7961 C > T	C	T	50%	*MT-ATP8/6*	TAA	Pro-Leu/Gln-STOP	ns/ns
	Positive	m.13224 G>A	G	A	50%	*MT-ND5*	ATA	Val-Met	ns
	Positive	m.15116 G>A	G	A	25%	*MT-CYB*	TTA	Leu-Leu	syn
2	Positive	m.2291 T>C	T	C	50%	*MT-16SrRNA*			-
3	Positive	m.5957 A>G	A	G	25%	*MT-CO1*	CTG	Leu-Leu	syn

Crypt	COX Histochemistry	Mutation	WT base	Mutant base	Heteroplasmy level	Gene	Codon change	Amino acid change	Substitution type
	Positive	m.12723 C>T	C	T	25%	*MT-ND5*	TAC	His-Tyr	ns
	Positive	m.12783 C>T	C	T	75%	*MT-ND5*	TAT	His-Tyr	ns
4	Positive	m.3084T>C	T	C	25%	*MT-ND1*	CCA	Ser-Pro	ns
	Positive	m.8578 G>A	G	A	75%	*MT-ATP6*	ATA	Val-Met	ns
	Positive	m.11970 A>G	A	G	75%	*MT-ND5*	GAA	Lys-Glu	ns
	Positive	m.12783 C>T	C	T	50%	*MT-ND5*	TAT	His-Tyr	ns
	Positive	m. 13200 A>T	A	T	50%	*MT-ND5*	TAA	Lys-STOP	ns
	Positive	m.15164 C>T	C	T	50%	*MT-CYB*	GGT	Gly-Gly	syn
5	Positive	m.381 A>T	A	T	75%	*MT-16SrRNA*			-
	Positive	m.6265 C>T	C	T	50%	*MT-CO1*	GTA	Ala-Val	ns
	Positive	m.7860 C>T	C	T	25%	*MT-ATP8*	ATA	Thr-Met	ns
	Positive	m.8079 A>T	A	T	50%	*MT-ATP6*	AAT	Lys-Asn	ns
	Positive	m.8126 C>T	C	T	50%	*MT-ATP6*	ATA	Thr-Met	ns
	Positive	m.10717 A>T	A	T	50%	*MT-ND4*	CTC	His-Leu	ns
	Positive	m.12105 A>T	A	T	75%	*MT-ND5*	TCA	Thr-Ser	ns
	Positive	m.12353 C>T	C	T	75%	*MT-ND5*	TCT	Ser-Ser	syn
	Positive	m.12592 C>T	C	T	75%	*MT-ND5*	ATC	Thr-Ile	ns
	Positive	m. 14658 A>T	A	T	75%	*MT-CYB*	TAA	Lys-STOP	ns
6	Positive	m.3541 T>C	T	C	50%	*MT-ND1*	CCA	Leu-Pro	ns
	Positive	m.5966 C>T	C	T	25%	*MT-CO1*	CGT	Arg-Arg	syn
	Positive	m.8048 G>A	G	A	25%	*MT-ATP6*	CAT	Arg-His	ns
	Positive	m.8489 C>T	C	T	50%	*MT-ATP6*	ATA	Thr-Met	ns
	Positive	m.11001 C>T	C	T	25%	*MT-ND4*	TAA	Gln-STOP	ns
	Positive	m.11113 T>C	T	C	50%	*MT-ND4*	ACA	Met-Thr	ns
	Positive	m.11180 C>T	C	T	25%	*MT-ND4*	CAT	His-His	syn
	Positive	m.13403 C>T	C	T	75%	*MT-ND5*	GAT	Asp-Asp	syn

Crypt	COX Histochemistry	Mutation	WT base	Mutant base	Heteroplasmy level	Gene	Codon change	Amino acid change	Substitution type
7	Positive	m.3908 G>A	G	A	50%	*MT-tRNA M*	A:G Mismatch to A:A in AA stem		-
	Positive	m.8518 C>A	C	A	75%	*MT-ATP6*	ATA	Leu-Met	ns
	Positive	n.12088 G>T	G	T	25%	*MT-ND5*	CTA	Arg-Leu	ns
	Positive	m.12211 G>A	G	A	25%	*MT-ND5*	TAA	Trp-STOP	ns
8	Positive	m.13033 C>T	C	T	25%	*MT-ND5*	ATA	Trp-Met	ns
9	Positive	m.5499 G>A	G	A	50%	*MT-CO1*	ATA	Val-Met	ns
	Positive	m.8065 C>T	C	T	25%	*MT-ATP6*	TAC	His-Tyr	ns
	Positive	m.13435 C>T	C	T	25%	*MT-ND5*	TTC	Ser-Phe	ns
10	Positive	m.2525 C>T	C	T	25%	*MT-16SrRNA*			-
	Positive	m.3226 C>T	C	T	75%	*MT-ND1*	TTC	Ser-Phe	ns
	Positive	m.5668 C>T	C	T	75%	*MT-CO1*	GTA	Ala-Val	ns
	Positive	m.6960 A>G	A	G	50%	*MT- tRNA D*	DHU Loop		-
	Positive	m.7881 C>T	C	T	25%	*MT-ATP8*	CTA	Pro-Leu	ns
	Positive	m.8955 C>T	C	T	25%	*MT-CO3*	TCT	pro-Ser	ns
	Positive	m.9780 C>T	C	T	75%	*MT-ND3*	TAA	Gln-STOP	ns
	Positive	m. 10549 C>T	C	T	25%	*MT-ND4*	CTA	Pro-Leu	ns
	Positive	m.10939 C>T	C	T	75%	*MT-ND4*	GTA	Ala-Val	ns
	Positive	m.11066 A>T	A	T	25%	*MT-ND4*	TCT	Ser-Ser	syn
	Positive	m.12942 A>T	A	T	75%	*MT-ND5*	TCC	Thr-Ser	ns
11	Positive	m.10466 C>T	C	T	50%	*MT-ND4*	ATT	Ile-Ile	syn
	Positive	m.14447 C>T	C	T	75%	*MT-CYB*	GGT	Gly-Gly	syn
	Positive	m.15191 C>T	C	T	75%	*MT-CYB*	ATT	Ile-Ile	syn
	Positive	m.15202 T>C	T	C	75%	*MT-CYB*	CCA	Leu-Pro	ns
12	Positive	none	none	none	none	none	none	none	none
