# Supplementary material for: Similar patterns of clonally expanded somatic mtDNA mutations in the colon of heterozygous mtDNA mutator mice and ageing humans
Source: Mech Ageing Dev. 2014 Jul;139:22–30. doi: 10.1016/j.mad.2014.06.003 (PMC4141908; doi:10.1016/j.mad.2014.06.003)
Supplement: Supplementary File 3 — Table comparing the location, types and frequency of mtDNA point mutations in colonic crypts from PolgA+/Mut mice and aged humans. [file mmc3.pdf]

	Mutation frequency in COX +ve crypts		Mutation frequency in COX -ve crypts	
	PolgA$^{+/mut}$ mice	Humans	PolgA$^{+/mut}$ mice	Humans
Transitions	44 (80%)	28 (85%)	150 (81%)	82 (86%)
Transversions	11 (20%)	2 (6%)	36 (19%)	5 (5%)
Ins/Dels	0	3 (9%)	0	9(9%)
Complex 1	27 (49%)	8 (24%)	64 (34%)	23 (24%)
Complex 3	6 (11%)	1 (3%)	14 (8%)	7 (7%)
Complex 4	6 (11%)	9 (27%)	51 (27%)	29 (30%)
Complex 5	10 (18%)	0	4 (2%)	2 (2%)
non-coding RNA	6 (11%)	9 (27%)	47 (25%)	26 (27%)
non-coding mtDNA	0	6 (18%)	6 (3%)	9 (9%)

Supplementary File 3: Table comparing the location, types and frequency of mtDNA point mutations in colonic crypts from *PolgA* $^{+/Mut}$ mice and aged humans (Greaves et al 2012). Ins/Dels - Insertions and deletions
