# Supplementary material for: Similar patterns of clonally expanded somatic mtDNA mutations in the colon of heterozygous mtDNA mutator mice and ageing humans
Source: Mech Ageing Dev. 2014 Jul;139:22–30. doi: 10.1016/j.mad.2014.06.003 (PMC4141908; doi:10.1016/j.mad.2014.06.003)
Supplement: Supplementary File 4 — Respiratory chain deficiency in ageing PolgAmut/mut mouse colon. [file mmc4.pdf]

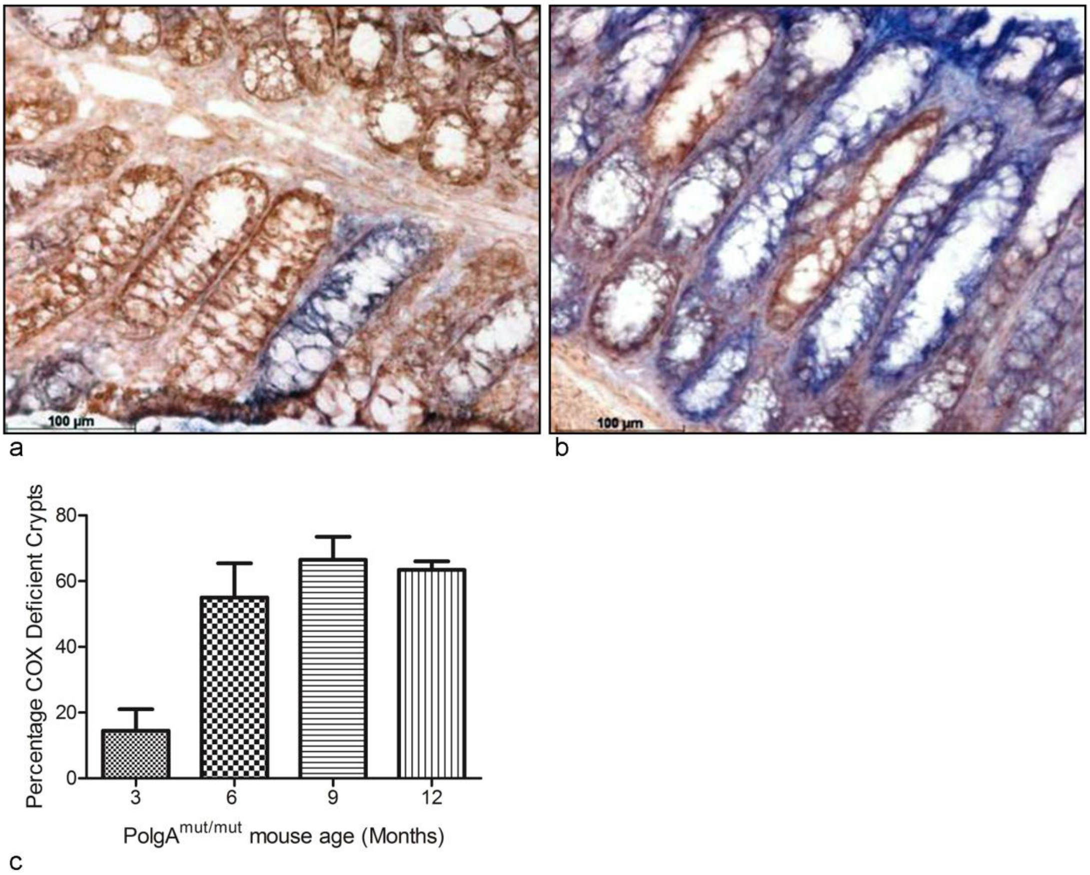

Supplementary File 4: Respiratory chain deficiency in ageing *PolgA*$^{mut/mut}$ mouse colon. (**a**) COX/SDH histochemistry on 3 month old *PolgA*$^{mut/mut}$ mouse colon. (**b**): COX/SDH histochemistry on 12 month old *PolgA*$^{mut/mut}$ mouse colon. Crypts stained brown are positive for COX activity, those stained blue are COX deficient and those stained purple/grey display intermediate COX deficiency. (**c**): Mean incidence (± SEM) of COX deficient colonic crypts in 3, 6, 9 and 12 month old *PolgA*$^{mut/mut}$ mice.
