# Supplementary material for: Similar patterns of clonally expanded somatic mtDNA mutations in the colon of heterozygous mtDNA mutator mice and ageing humans
Source: Mech Ageing Dev. 2014 Jul;139:22–30. doi: 10.1016/j.mad.2014.06.003 (PMC4141908; doi:10.1016/j.mad.2014.06.003)
Supplement: Supplementary File 5 — Description of in silico modelling of clonal expansion of mtDNA mutations. [file mmc5.pdf]

Supplementary File 5: Model of mtDNA mutation clonal expansion by random genetic drift

The model was developed in MATLAB and describes the accumulation of mutated mtDNA molecules contributing to overall COX deficiency within a rapidly dividing stem cell lineage. It was used to investigate how the rate at which new mtDNA mutations are introduced into the mtDNA pool (mutRate) affects the frequency of observed mtDNA mutations. 1000 cells were simulated for each mtDNA mutation rate.

The model simulates the replication and segregation of mtDNA molecules according to the relaxed replication and random segregation of mtDNA molecules hypothesis that has been suggested within the literature (1-3). The model tracks the number of mutated mtDNA molecules of a dividing stem cell for every division that occurs.

Replication

When a stem cell undergoes division, the number of mtDNA molecules has to be doubled in order for there to be a constant number of mtDNA molecules within each daughter cell. Therefore, within the model there is a replication stage in order to attain mtDNATot*2 number of mtDNA molecules before division. As mtDNA molecules are either in the mutated or non-mutated state, a replication probability for each replication has to be determined to attain what the probability is of replicating a mutated mtDNA molecule. This is done sequentially for each mtDNA replication (RepNo) until mtDNATot*2 molecules have been generated. The equation for this is as follows:-

$$RepProb = \frac{mtDNAMut}{(mtDNATot + RepNo - 1)}$$

Equation 1: mtDNAMut is the number of mtDNA molecules within the stem cell that have a mutation contributing to COX deficiency, mtDNATot is the total number of mtDNA molecules within a cell before division, RepNo refers to how many new mtDNA molecule have been generated. For example, when there is 1 mutated mitochondrial molecule present in the cell of a total of 50, the probability that this mtDNA molecule is replicated at the first replication event is 1/50, the second replication event, 1/51, the last replication event, 1/99. A mutated mtDNA

molecule is only replicated when the random number that is generated is less than the value of RepProb at that time.

As new mtDNA molecules need to be generated, the replication probability changes with each consecutive replication. A random number between 0 and 1 is generated for each mtDNA that is replicated. If the probability of replication (RepProb) is less than the random number generated, the number of mutated mtDNA molecules is increased by one. If normal mtDNA molecules are chosen for replication, they may become mutated through replication errors and are subjected to a mutation rate. Another random number is generated and if that number is less than the mutRate, the number of mutated mtDNA molecules increases by one. Once mtDNATot*2 have been generated they then need to randomly segregate into a single daughter cell.

Segregation

The chance of one of the daughter cells receiving a mutated mtDNA molecule is dependent on a division probability (DivProb). This is done sequentially for each mtDNA molecule segregating into a daughter cell until 'mtDNATot' number of mtDNA molecules have been segregated. The number of mutated mtDNA molecules segregated into the daughter cell is tracked via the NewCell variable in order to have a correction for the number of mutated mtDNA molecules that have already been segregated into the daughter cell

$$DivProb = \frac{(mtDNAMut - NewCell)}{(2 * mtDNATot) - (DivNo - 1)}$$

Equation 2: mtDNAMut is the number of mtDNA molecules within the stem cell that have a mutation contributing to COX deficiency, NewCell is the number of mutated mtDNA molecules that have already been segregated into the daughter cell, mtDNATot is the total number of mtDNA molecules within a cell before division, DivNo refers to how many mtDNA molecules have been segregated into one of the daughter cells. For example, when there is 1 mutated mitochondrial molecule present in the dividing stem cell of a total of 50 mtDNA molecules before division, the probability that this mtDNA molecule is segregated into a daughter cell is

1/100, the second segregation event, 1/99, the last segregation event, 1/51. A mutated mtDNA molecule is only segregated when the random number that is generated is less than the value of DivProb at that time.

Once all the mtDNA molecules have been segregated, the NewCell variable replaces the mtDNAMut variable in the next replication and division cycle and the number of mutated mtDNA molecules is tracked throughout the lifetime of the stem cell's stem cell progeny.

1. Coller, H.A., Khrapko, K., Bodyak, N.D., Nekhaeva, E., Herrero-Jimenez, P. and Thilly, W.G. (2001) High frequency of homoplasmic mitochondrial DNA mutations in human tumors can be explained without selection. *Nat Genet*, **28**, 147-150.
2. Elson, J.L., Samuels, D.C., Turnbull, D.M. and Chinnery, P.F. (2001) Random intracellular drift explains the clonal expansion of mitochondrial DNA mutations with age. *Am J Hum Genet*, **68**, 802-806.
3. Taylor, R.W., Barron, M.J., Borthwick, G.M., Gospel, A., Chinnery, P.F., Samuels, D.C., Taylor, G.A., Plusa, S.M., Needham, S.J., Greaves, L.C. *et al.* (2003) Mitochondrial DNA mutations in human colonic crypt stem cells. *J Clin Invest*, **112**, 1351-1360.
